# COVID-19 adaptations to a training and support programme to improve primary care response to domestic abuse: a mixed methods rapid study

**DOI:** 10.1186/s12875-023-02203-5

**Published:** 2024-01-10

**Authors:** Lucy Downes, Estela Capelas Barbosa

**Affiliations:** 1 IRISi, 10 Park Street, BS1 5HX Bristol, UK; 2 Bristol Medical School, University of Bristol, Canynge Hall, BS8 2PN Bristol, UK

**Keywords:** Domestic abuse, Primary care, Training, Advocacy support, Mixed methods, COVID-19.

## Abstract

**Background:**

Increased incidence and/or reporting of domestic abuse (DA) accompanied the COVID-19 pandemic. National lockdowns and enforced social isolation necessitated new ways of supporting victims of DA remotely. ***I***dentification and ***R***eferral to ***I***mprove ***S***afety (IRIS) is a programme to improve the response to domestic abuse in general practice, providing training for general practice teams and support for patients affected by DA, which has previously been proven effective and cost-effective [1–3]. The COVID-19 pandemic required the adaptation of the programme to online training and remote support.

**Methods:**

This study is mixed methods rapid research, which aimed to gather evidence around the relevance, desirability and acceptability of IRIS operating remotely. Quantitative IRIS referral data were triangulated with data from four surveys and 15 interviews. Participants were local IRIS teams, IRIS-trained clinicians, and victim-survivors supported by IRIS services. The study was designed using the Lean Impact approach, allowing quick evaluation of innovation and the impact of social interventions. We carried out a framework analysis of the interviews, which is a qualitative methodology widely used in policy and applied research that enables research teams to move from descriptive accounts to a conceptual explanation of findings [4, 5].

**Results:**

We found that the adaptation to online training and support of IRIS was acceptable and desirable. Most clinicians felt confident addressing DA over the phone and online, although most were more confident face-to-face. While referrals to IRIS services initially declined in March 2020, numbers of referrals increased to pre-pandemic levels by July 2020. Patients felt well supported remotely, although patients who had previously experienced face-to-face support preferred it. Technology was the most frequently mentioned barrier to the change from face-to-face training and support to online training and remote support.

**Conclusions:**

This study contributes to practice by asserting the desirability and acceptability of training clinicians to be able to identify, ask about DA and refer to the IRIS programme during telephone/online consultations. This is of relevance to health and public health commissioners when making commissioning decisions to improve the general practice response to domestic abuse.

**Supplementary Information:**

The online version contains supplementary material available at 10.1186/s12875-023-02203-5.

## Background

Domestic abuse (DA) is a breach of human rights and a major public health problem with devastating health consequences and enormous costs to health services [6]. In the UK alone, an estimated 9 million women (27.6%) and 4.6 million men (13.8%) experience domestic abuse during their adult lifetime [7]. The estimated financial burden of DA to the NHS is £2.3 billion per year [8]. The health consequences of experiencing DA are wide-ranging, profound and long-lasting [9–11]. The prevalence of DA amongst female patients accessing their general practice is significantly higher than the UK average [12], so general practice can play an essential role in responding to and helping to prevent DA by intervening early, providing treatment and information and referring patients to specialist services.

Some health professionals are confident and competent in asking about and responding to disclosures of domestic abuse, but many lack confidence, and are frequently unaware of referral pathways to appropriate specialist support services [13, 14]. ***I***dentification and ***R***eferral to ***I***mprove ***S***afety (IRIS) is a programme of training and support to improve the general practice response to domestic abuse. The programme trains clinicians to identify and respond to patients (primarily women) affected by DA and offer a referral to a specialist, named IRIS Advocate Educator (AE) based in a third sector DA agency (an IRIS partner organisation) and embedded within participating practices. Patient-led advocacy and support is provided to referred patients by the IRIS AE.

The IRIS Programme is considered the gold standard in primary care responses to DA [15–17]. It was evaluated in a pragmatic cluster randomized control trial (RCT) and in post-implementation settings. The RCT saw a six-fold increase in referrals to specialist DA services by IRIS-trained general practices [18], and demonstrated the intervention to be cost-effective [19] and acceptable to clinicians [20] and patients [21]. Post-trial evaluations of IRIS implementation in the real-world showed referral rates and acceptability remained consistent with the original trial [22, 23] and that the programme remained cost-effective [24]. The evidence-based IRIS Programme design featured face-to-face training for healthcare staff and, largely, face-to-face advocacy and support for referred patients, frequently provided on practice premises. Identification of patients affected by DA took place in consultations which were predominantly conducted face-to-face.

The COVID-19 pandemic saw an increase in the global incidence and reporting of DA [25, 26]. National lockdowns alongside social distancing and self-isolation required a sudden and widespread shift in the provision of consultations in general practice from face-to-face to phone/video consultations [27]. In theory, excepting the first few weeks from 23rd March 2020 when all general practices were closed, general practitioners could continue to address domestic abuse in consultations (regardless of the consultation method). In practice, however, losing the assured confidentiality of face-to-face consultations may have raised concerns about whether it was safe to ask about domestic abuse. The patient may not have been alone; the perpetrator may have been present or able to overhear. Secondly, body language cues became limited or non-existent by video/phone, making it more difficult for clinicians to accurately gauge a situation or a patient’s response. This sudden shift resulted in the need to provide (virtually) new training and guidance for general practice clinicians focussed on safely addressing domestic abuse in remote consultations, and also new ways of providing support (remotely) to patients referred into the IRIS Programme [28].

## Methods

### Aim

This study intended to provide initial evidence in response to three questions:

1. Could confidentiality (required for safe disclosures of DA) be guaranteed in remote consultations?
2. Was IRIS training effective and acceptable to clinicians when delivered virtually?
3. Was IRIS advocacy support effective and acceptable to patients when delivered remotely?

### Study design

A Rapid Research study using a mixed methods design was conducted. We used a Lean Impact approach, intended to quickly evaluate innovation and the impact of social interventions [29]. This approach was selected to provide prompt assessment of the swift adaptations necessary to respond to the pandemic restrictions on social interaction.

The Lean Impact approach encourages innovators to create three sets of hypotheses: (a) a value hypothesis, which tests whether an intervention is desirable and embraced by relevant stakeholders; (b) a growth hypothesis, which tests whether the intervention can be upscaled to meet need and produce economies of scale, and (c) an impact hypothesis, which tests the intervention’s effectiveness. Our focus was on (a) and (c). We did not generate a growth hypothesis because, although initial scaling success had already been demonstrated (from 2 areas included in the RCT to approximately 33 areas by 2020), we did not consider at the time that it would be possible to further scale the IRIS Programme during the pandemic and accompanying restrictions. To understand whether the IRIS Programme remained desirable, acceptable and effective when delivered remotely, we explored three different hypotheses. Workstream 1 explored confidentiality of consultations in general practice - in large part taking place by phone/video since pandemic restrictions were introduced – and the relevance of this to identifying and responding to domestic abuse. Workstream 2 explored the desirability and acceptability of conducting IRIS training online for AEs, Clinical Leads (CLs) and clinicians. Workstream 3 explored the desirability and acceptability of remote/online/phone advocacy for patients/service users. Table 1 summarises the hypotheses for each workstream.

**Table 1:** Lean Impact approach: Hypotheses and workstreams

	Hypothesis	How to test this hypothesis
WS1	Assured confidentiality of consultations is critical to identifying and referring patients affected by domestic abuse. It is harder to make referrals to IRIS since lockdown restrictions were introduced.	Compare referral numbers pre and post the implementation of COVID-19 social distancing restrictions. Survey clinicians on their confidence and ability to discuss DA during telephone consultations. Interview patients on their confidence and ability to respond to questions about/raise DA during telephone consultations.
WS2	Clinicians / AEs / CLs find online IRIS training effective and desirable, although potentially less so than face-to-face training	Survey followed by sample interviews with clinicians, AEs and CLs
WS3	Patients find online/remote advocacy effective and desirable, although potentially less so than face-to-face advocacy	Sample interviews with patients.

### Setting and participants

IRISi is a social enterprise established to improve the healthcare response to gender-based violence. It functions as a hub organisation supporting commissioning and implementation of the IRIS Programme. It maintains contact with and between local IRIS team members in areas where IRIS is commissioned (IRIS sites); collectively this comprises the IRIS Network. The DA agencies delivering the IRIS Programme in IRIS sites (IRIS Partner organisations) provide IRISi with quarterly anonymised quantitative data on referrals received from IRIS-trained practices (referral data). We followed the UN Women decision tree for collecting data on violence against women and girls and COVID-19 [30]. Consequently, we used participants who we could safely recruit through the IRIS Network as a convenience sample. Local IRIS team members (AEs and CLs) were eligible to participate in Workstream 2. Additional participants for Workstreams 1, 2 and 3 (IRIS-trained clinicians and patients supported by local IRIS services) were recruited by AEs functioning as gatekeepers. Anyone who either did not consent or did not respond was excluded. Whilst acknowledging the limitations of using a convenience sample we justified this due to the rapid research method in which learning and iterating quickly are prioritised over extensive planning and research [31].

### Data collection: surveys and interviews

To allow for triangulation, workstreams 1 and 2 relied on more than one data collection method. Workstream 1 relied on quantitative (referral) data analysis before and after the United Kingdom went into the first national lockdown, exploring the period between January and July 2020. Additionally, we surveyed clinicians in IRIS-trained practices and interviewed patients/service users to understand their confidence and ability to discuss and/or disclose DA in remote consultations. Workstream 2 surveyed clinicians, CLs and AEs about their perceptions of the acceptability and desirability of online training. These views were explored in more depth during the interviews. Workstream 3 relied on semi-structured interviews with service users. Originally, we had also planned to collect pre and post feedback from patients/services users for Workstream 3. However, the pre pandemic feedback forms we had available for analysis did not include questions on confidentiality in remote consultations and the effectiveness and desirability of remote support. Consequently, the patient feedback forms were deemed inappropriate for our hypotheses, meaning Workstream 3 findings are based on the conducted interviews and on some questions in the AE survey. Surveys were administered during July and August 2020 and the interviews were conducted during August 2020. The surveys were provided in English only, and the interviews were conducted in English only. Survey responses were anonymous.

Surveys for AEs and CLs were sent to the entire IRIS Network (comprising of 187 people). 56 responded. AEs were invited to forward the survey for IRIS-trained clinicians to their local IRIS-trained practices. The total number of clinicians to whom the survey was forwarded is unknown, thus a response rate cannot be calculated. One hundred and fifteen clinicians responded.

We defined a convenience sample of 15 individuals (5 AEs, 3 CLs, 3 clinicians and 4 service users) to interview. The surveys for AEs, CLs and clinicians invited respondents to provide their contact details if they were willing to be interviewed. A selection of those who provided their details were invited to be interviewed. A range of participants from new/longstanding sites and rural/urban locations were chosen to ensure varied perspectives. AEs were invited to consider whether any patients they had supported remotely might be willing to be interviewed, to discuss this invitation with those service users (if safe to do so) and to pass on the service users’ contact details to the researchers if the service user consented. While the number of volunteers was larger than the target sample size for AEs, CLS and clinicians, only four service users volunteered in the two-week recruitment window so all four were interviewed. An information sheet was provided to each participant (appendix 2). Each participant gave their continued informed consent to take part. We recognised that although the target sample size was relatively small, it would provide enough data to apply the Lean Impact method and the framework analysis. Ethical approval for the study was waived by the University of Bristol Faculty of Health Science Research Ethics Committee, which deemed the study a service evaluation.

### Analysis

Survey data and referral data were analysed using descriptive statistics. We carried out a framework analysis to understand overarching patterns arising from interviews with multiple stakeholders. We selected this method for its systematic and flexible approach to analysing qualitative data, appropriate for use in research teams - like ours - where some members have limited experience of conducting qualitative research [32]. We used a simple matrix as our analytical framework, exploring the themes “Barriers”, “Enablers”, “Losses” and “Benefits” during analysis. All interviews were analysed by two independent researchers. Disagreements in coding were discussed until consensus was reached.

## Results

Results are presented by data collection format, beginning with referral data results, followed by survey data results, and thirdly interview data results.

### Referral data

The referral data are relevant to Workstream 1, which explored potential difficulties of ensuring confidentiality when seeking to identify domestic abuse during remote consultations. To make visible the impact of moving to remote consultations on clinicians’ ability to identify and refer patients affected by DA we compared daily referrals from January 2020 to July 2020.

**Fig. 1:**
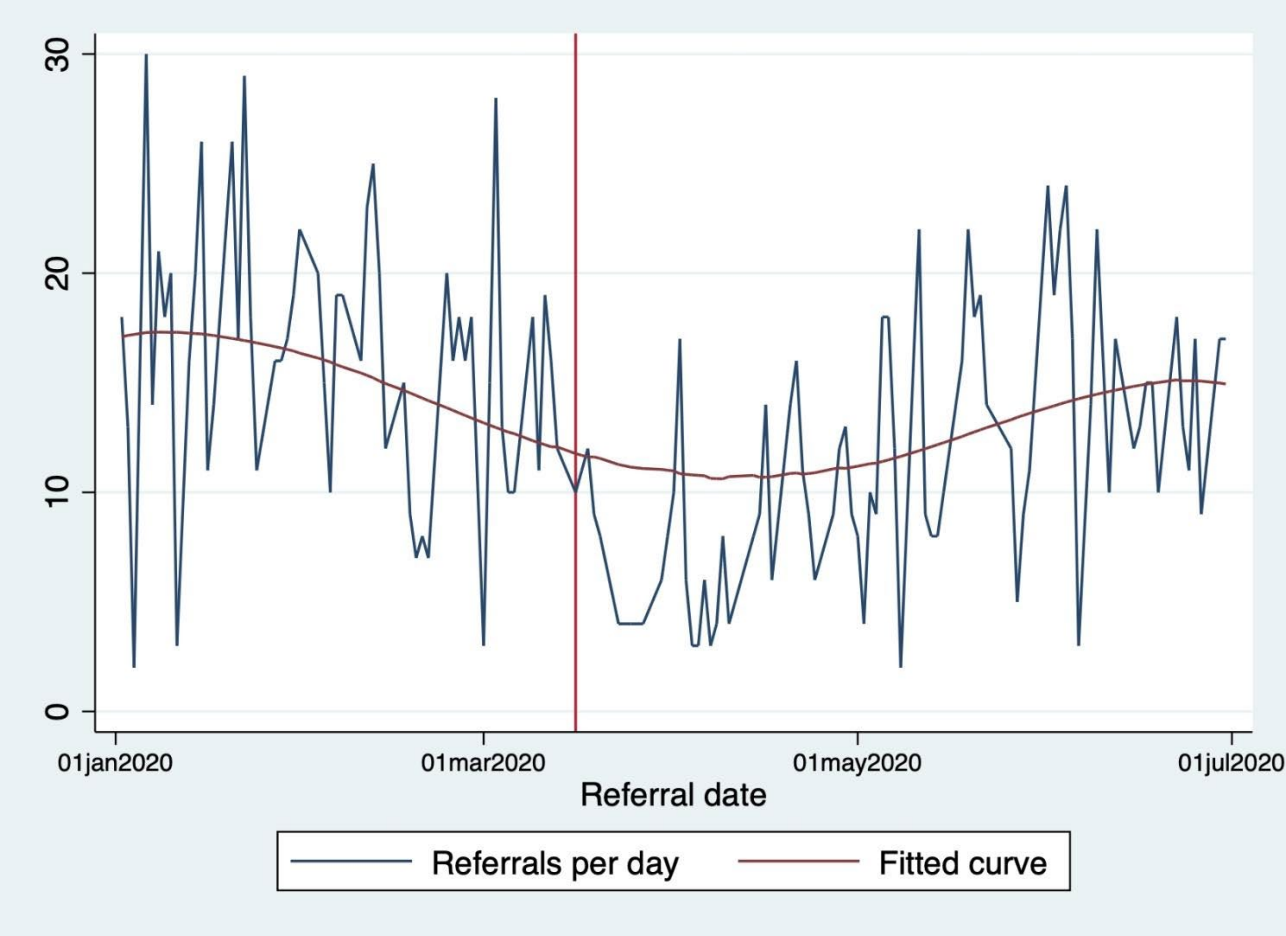
Daily referrals to IRIS services – January to July 2020

Figure 1 shows daily referrals from 1 January to 1 July 2020. After an initial decline in late March /early April, referrals began increasing again in late April. By July 2020, referral numbers had nearly reached pre-pandemic levels. While the long-term impact was outside the scope of this study, an interrupted time series analysis of the impact of the first COVID-19 national lockdown on referrals into IRIS services has been published elsewhere [33].

### Surveys

The survey data are relevant to Workstreams 1 (exploring potential difficulties in assuring the confidentiality of remote consultations) and 2 (exploring whether IRIS training provided online is effective and desirable). We used surveys to pursue several lines of enquiry with clinicians and CLs to explore how confident they felt to address domestic abuse during telephone/video consultations (and therefore to better understand the reasons for the sharp drop and gradual rise in referrals, coinciding with the first lockdown). Tables 2 and 3 summarise the results.

**Table 2:** Confidence level in addressing DA in remote consultations

	Confidence level in identifying and asking about DA in phone/video consultations		Confidence level in responding to disclosures of DA in phone/video consultations	
	IRIS Clinical Leads (n. 25)	Clinicians (n.115)	IRIS Clinical Leads (n.25)	Clinicians (n.115)
**Very confident**	36% (n.9)	13.9% (n.16)	68% (n.17)	22.6% (n.26)
**Somewhat confident**	44% (n 11)	48.7% (n.56)	20% (n.5)	47.8% (n.55)
**Neither confident nor unconfident**	16% (n.4)	21.7% (n.25)	12% (n.3)	19.1% (n.22)
**Somewhat unconfident**	4% (n.1)	14.8% (n.17)	0% (n.0)	9.6% (n.11)
**Very unconfident**	0% (n.0)	0.9% (n.1)	0% (n.0)	0.9% (n.1)
*t-test*	p = 0.076		p = 0.101	

**Table 3:** Confidence level addressing DA in remote consultations *compared with* confidence addressing DA in face-to-face consultations

	Identifying and asking about DA		Responding to disclosures of DA	
	Clinical Leads (n.25)	Clinicians (n.115)	Clinical Leads (n.25)	Clinicians (n.115)
**I am much more confident in face-to-face consultations**	32% (n.8)	37.4% (n.43)	20% (n.5)	30.4% (n.35)
**I am somewhat more confident in face-to-face consultations**	28% (n.7)	44.3% (n.51)	20% (n.5)	33.9% (n.39)
**I have the same level of confidence for both face-to-face consultations and phone/video consultations**	28% (n.7)	13% (n.15)	60% (n.15)	34.8% (n.40)
**I am somewhat less confident in face-to-face consultations**	12% (n.3)	4.3% (n.5)	0% (n.0)	0.9% (n.1)
**I am much less confident in face-to-face consultations**	0% (n.0)	0.9% (n.1)	0% (n.0)	0% (n.0)
*t-test*	p = 0.115		p = 0.069	

Table 2 illustrates that four in five (80%, 20 of 25) CLs, and nearly two thirds (62.6%, 72 of 115) of clinicians felt confident identifying and asking about DA during telephone/video consultations. A key concern prompting this rapid study had been that the lack of assured confidentiality of remote consultations may lead fewer clinicians to feel confident in identifying and asking about domestic abuse, although they may still feel confident responding to unprompted disclosures. Consequently, we differentiated between *actively identifying and asking* about DA as opposed to *responding* to a disclosure. As hypothesised, a slightly greater proportion of both CLs (88%, 22 of 25) and clinicians (70.4%, 81 of 115) felt confident *responding* to disclosures of DA during telephone/video consultations. We did not disaggregate outcomes for addressing and responding to DA between video and telephone consultations as both may pose risks around confidentiality.

Additionally, we asked clinicians and CLs how their confidence in (a) identifying and asking and (b) responding to disclosures of DA in remote consultations compared with their confidence in addressing DA in face-to-face consultations. Table 3 summarises the results.

We had hypothesised that clinicians and CLs would feel more confident addressing DA in face-to-face consultations, where they could be assured of the confidentiality (and therefore the patient’s safety) of asking about DA. As predicted, the majority of CLs (60%, 15 of 25) and clinicians (81.7%, 94 of 115) felt more confident identifying and asking about DA face-to-face. Nearly two thirds of CLs and over three quarters of clinicians feel less confident to ask about DA in remote consultations. In addition, the majority of clinicians (64.1%, 74 of 115) also felt more confident to respond to disclosures of DA in face-to-face consultations (and logically less confident to respond remotely), as did 40% (10 of 25) of CLs.

Finally, we surveyed clinicians on whether they had actively asked about domestic abuse in phone/video consultations: 61.7% of clinicians (71 of 115) responded that they had actively asked about DA in remote consultations. 48.7% of clinicians (56 of 115) reported responding to a disclosure remotely and 33.9% (39 of 115) had made a referral to an IRIS AE during a phone/video consultation. This indicates that, despite lack of confidence, clinicians are still making attempts to actively identify and ask about domestic abuse during remote consultations, and nearly half had indeed received a disclosure from a patient, thereby demonstrating the relevance and importance of asking.

We used surveys to explore whether our different groups of stakeholders (AEs, clinicians, and CLs) found it acceptable and desirable for IRIS training to be delivered virtually. This was part of Workstream 2. Unfortunately, during the data collection period (August 2020), only 4.3% of clinicians (5 of 115) who responded had yet received IRIS training online so the results presented are largely the views of AEs and CLs. By March 2022, the proportion of clinicians receiving training online had grown substantively, with more than 87.9% of clinicians (751 of 855) having received IRIS training online (snapshot taken from IRISi’s internal database, 7th March 2022).

Our AE survey (39 respondents) revealed mixed perceptions around online training. Nearly half of respondents (48.7%, 19 of 39) felt that clinicians would engage better with IRIS training in its current online form due to the perceived increased relevance of the IRIS Programme following greater media coverage of DA, and a quarter (25.6%, 10 of 39) believed clinicians would find it easier to commit time for online training, particularly while fewer patients seeking access to general practice had reduced workload. Despite this, nearly half (48.7%, 19 of 39)^1^ of respondents were concerned about difficulties in concentration, engagement or IT/technological difficulties. Around one quarter (25.6%, 10 of 39) were concerned that online training would prove less effective, although only a minority (15.4%, 6 of 39) believed training a practice team online would result in fewer patients referred for advocacy and support.

CLs and clinicians were more positive and pragmatic about online training. Of the Clinical Leads surveyed, 72% of respondents (18 of 25) preferred online training to face-to-face. Although AEs expressed concern about maintaining clinicians’ engagement in online training, clinicians themselves have responded that they were able to engage and absorb online training well. Of the clinicians surveyed who had completed online IRIS training, 100% (5) found a quiet place to complete the training, they didn’t have technical difficulties, they managed to dedicate time for training without distractions and they found it straightforward to access materials before and after the training.

The AE survey included questions on the perceived effectiveness and desirability of IRIS remote advocacy and support. In the AE survey (39 respondents, although not every respondent answered every question), the majority (78.2%, 25 of 32) reported that all or most of their service users were happy to transfer from face-to-face to remote support. Only small minorities of AEs reported that most service users chose not to take up remote support (3.1%, 1 of 32), that most were unable to find a safe/quiet/confidential space in which to engage with remote support (3.1%, 1 of 32), or that most were prevented from accessing remote support because of additional caring responsibilities (3.1%, 1 of 32). Similarly small percentages (3.1%, 1 of 32) reported that around half of service users could not engage with remote support because of lack of access to an interpreter, or lack of access to the necessary technology. These findings indicate that remote advocacy and support, when offered, is acceptable to the large majority of service users.

A more nuanced picture emerges about the desirability of remote support and its outcomes. A majority (71.8%) of AEs (28 of 39) reported that service users’ self-reported outcomes were just as positive about remote support as compared with past face-to-face support. Nearly half (46.2%) of AEs (18 of 39), however, reported delays or difficulties in access for their service users to survivor’s groups or other relevant services, broadly correlating with a sizeable minority of AEs (39%, 15 of 39) who reported that they felt something was lacking in the support they were able to provide. The frequency of topics raised in free text detail on what would have improved this perceived lack were counted and revealed two main trends: better access to technology (for either themselves or their service users), and/or feeling better equipped to provide intensive emotional or therapeutic support, particularly in the temporary absence of many other services (such as counselling or group therapy) functioning.

The majority (71.8%) of AEs (28 of 39) reported providing additional emotional support to service users, and 41% (16 of 39) provided additional advice and information on legal, welfare and housing services.

### Semi-structured interviews

We used semi-structured interviews to better understand how confident (or not) clinicians felt to address DA in phone/video consultations (this was part of Workstream 1). The interviews revealed that concerns around safety contributed to this lack of confidence, as *“You can never be 100% sure she’s on her own”* (IRIS CL). The interviews also revealed a greater difficulty establishing rapport contributes to hesitancy to ask about DA:

“If you’ve never seen that patient before, got no relationship, then again I think that’s one of the factors that makes me hesitant to say in that first consultation whereas if I’d seen her face-to-face, I would’ve got that connection straight away and I would’ve asked her. There’s definitely barriers doing it virtually.” *IRIS CL*.

The third challenge to asking about DA in phone consultations was the difficulty of asking sensitive questions without the nuance and responsiveness of facial expressions and body language:

"You can’t see how the patient’s interpreting what you’re saying because everything you say is open to interpretation, isn’t it? Especially if they’re in a bad place." *IRIS CL*.

We used interviews to explore in more depth our stakeholders’ views on the effectiveness and desirability of online training. CLs voiced a preference for online training because blocking out full days is very difficult for clinicians. More concise sessions, as can be provided online, fit better with clinicians’ other commitments. Secondly, the CLs interviewed all highlighted that online training enabled IRIS teams to offer training more efficiently by training multiple practices in different locations at the same time. Thirdly, CLs raised that online sessions may make it possible to provide practice teams with more regular training, which would be beneficial given the frequency with which new COVID-19 guidance was being published. The following quote illustrates:

“We can offer an online meeting for anybody else across the other 60 practices or even the 90, who wants to come and just do an hour or an hour and a half update about IRIS in COVID-19. Actually, having done that, we should go on doing it year after year, because there are about 10 things that have changed in the last six months.” *IRIS CL*.

Clinicians reported feeling comfortable with online training and believed that in the near future no face-to-face trainings would be possible:

"I’m just trying to make sure that the program is still running in the best ability that it can be with lockdown and the use of the tele-communications but I think it’s also just to make sure that the online training still goes ahead for the GPs." *IRIS trained clinician.*

These findings indicate that online IRIS training can be desirable to clinicians in the context of the pandemic and perhaps more generally.

Interviews did also reveal some negative views on the effectiveness or desirability of online training. AE, CL and clinician participants consistently highlighted the difficulties of reading facial expressions and body language via a screen, with one participant describing this as “*talking into a void”* (IRIS CL). AE and Clinical Lead participants expressed concern about being unable to ascertain (or respond appropriately) if a training participant was distressed by the content because they could not see participants’ expressions. In contrast,

“...when you’ve got a room full of people, you can quite easily detect who’s struggling with the training or who it’s affecting.” *IRIS CL*.

We used interviews with service users to explore their views on being asked about DA during remote consultations (part of Workstream 1). Service users felt positive about being asked about domestic abuse, and offered a referral, during a remote consultation in general practice:

“I think it’s easier sometimes to do it on the phone because you wouldn’t hug a doctor when you’re crying anyway. I don’t mind it so much on the phone, the doctors.” *IRIS Service User.*

We used interviews to explore whether AEs (providing advocacy) and service users (receiving advocacy) found remote advocacy to be effective and desirable (Workstream 3). In interviews, both service users and AEs emphasised the responsiveness of AEs towards their service users. The quote illustrates:

"After we’ve had our phone call, she’s giving me a time and a date and if I needed to get hold of her, all I’d have to do was text and she answers really quick."

By providing more frequent contact and greater availability via text and Whatsapp, AEs ensured service users felt supported despite being unable to provide face-to-face advocacy. In interviews, service users stated that they felt their advocates had been able to accomplish things on their behalf:

“She’s done the referrals for social services. She got me on the Freedom Program. She did my legal aid for my solicitor, she helped him with his statements for court. She does do anything she can do remote. She does get it done." *IRIS Service User.*

As this quote demonstrates, service users perceived that remote advocacy can be highly effective.

Service users were interviewed about their preferences for either face-to-face or remote support. The two service users who had been able, pre-pandemic, to meet their advocate face-to-face preferred this. The two newer service users who had not had this option preferred remote support. This suggests that service users who are never offered face-to-face do not perceive a lack, and may indeed see it as more accessible:

“I know if remote wasn’t going on and it wasn’t available, I would just be missing an appointment because I don’t fancy going outside that day. I’m just in a low mood.” *IRIS Service User*.

Service users raised the relative ease of hiding emotions during remote advocacy sessions: *“it’s a lot easier to pretend you are happy on a video call and you’ve got your shit together”* (IRIS Service User). Perceptions on whether this is desirable were mixed. Some viewed this is as a barrier to connection, but others saw it positively. One service user explained how face-to-face support can feel overwhelmingly emotional, and so *“if I was going to cry, I’d rather do it on video at least”.*

Support for IRIS service users has always been described as ‘patient-led’. Our findings indicate that not only the type or topics of support but also the *mode* should be patient-led; advocacy cannot be provided using a one-size-fits-all approach.

### Framework analysis results

**Fig. 2:**
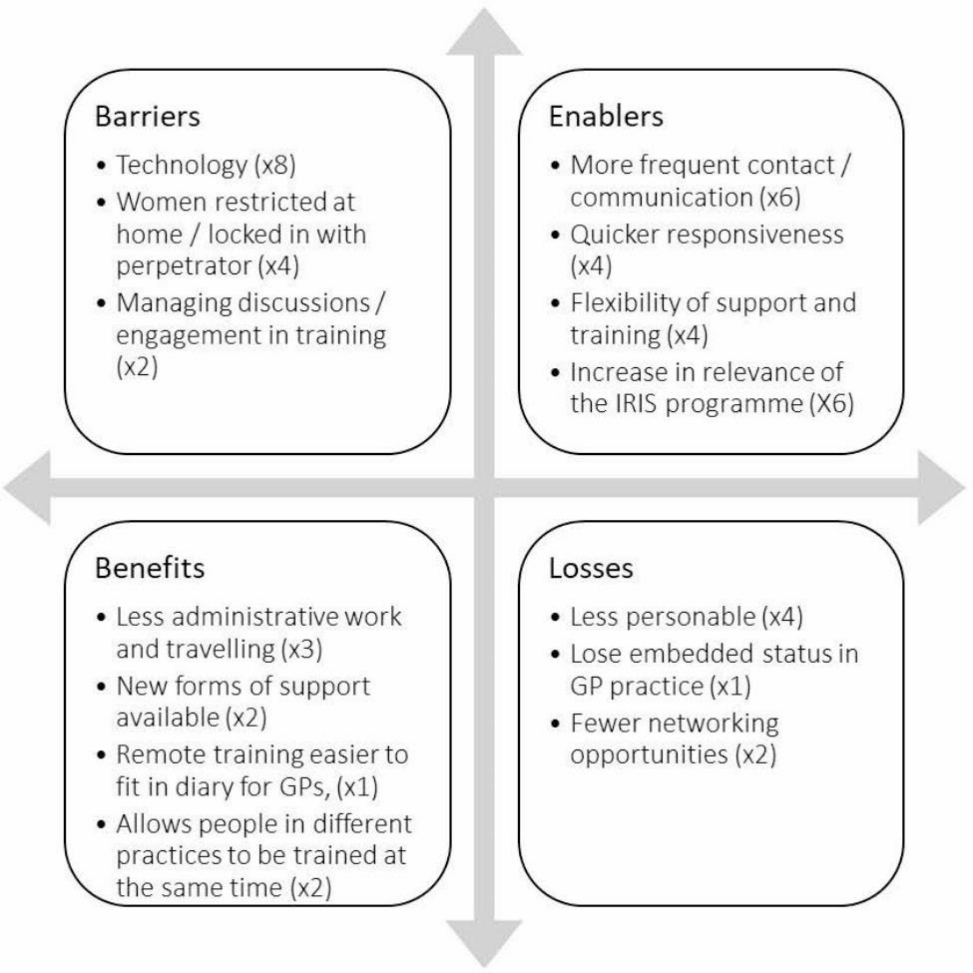
Framework analysis and number of interviewees mentioning common themes

We carried out a Framework analysis of the interviews focusing on barriers, enablers, benefits, and losses to understand commonalities and differences between AEs, CLs, clinicians and service users. Based on the analyses of the interviews, we produced a matrix of emerging findings, presented in Fig. 2. A similar matrix for each of the relevant groups of stakeholders in this research is provided in the supplementary materials.

The theme of technology as a (potential or actual) barrier was the most frequently mentioned across all stakeholders. Each type of stakeholder voiced concerns about technology as a barrier to the aspects of the IRIS Programme for which they are responsible for delivering or engaging with (e.g. AEs with delivering training and providing advocacy, clinicians with identifying abuse during consultations, etc.). Three themes of loss resonated across the different interviewee groups. Firstly, remote training and support is perceived as less personable, correlating with technology as a barrier. The second theme concerned the loss of the (physically) embedded status of the IRIS service and AE within IRIS-trained practices.^2^ Finally, AEs and CLs highlighted lost in-person networking opportunities.

Furthermore, four themes coalesced as enablers of change. Increased contact between different groups of stakeholders, combined with the speed and responsiveness of those delivering the IRIS Programme, and thirdly combined with greater flexibility of modes for training and support ensured that the IRIS Programme remained effective and desirable. The final enabler was the increase in relevance of the IRIS Programme in the context of increased prevalence and/or reporting of domestic abuse, compounded by usual routes to support being less or unavailable. Finally, the move to online training and remote support also produced benefits. The major themes, both for advocacy and training, were around increased time efficiency for those involved (less/no travel time means more sessions fit into busy working days), greater accessibility, and increased uptake as a result.

Comparing matrices a-d (found in the supplementary materials) illustrates that AEs and CLs (i.e. those delivering the IRIS Programme) more frequently discussed the barriers and enablers of the change from face-to-face to online/remote training and support, while clinicians and service users (the end beneficiaries of the IRIS Programme) focused more on the enablers of change.

## Discussion

In this study we have explored whether it was possible to ensure confidentiality of remote consultations to identify and refer patients affected by domestic abuse, if online IRIS training was effective and desirable for AEs, CLs and clinicians and if patients found online/remote advocacy effective and desirable. Our findings indicate the majority of clinicians felt more confident addressing DA in face-to-face consultations. While patient safety and assured confidentiality was indeed a key factor in this, our qualitative findings also indicated the increased difficulty in establishing rapport during remote consultations. Quantitatively, we found that there was a sharp decline in IRIS referrals immediately after the UK’s first national lockdown (March 2020), which could be an effect of closed practices as well as reduced confidence in referring, but by July 2020 referral numbers had nearly returned to those observed pre-pandemic.

Perceptions around the acceptability, desirability and effectiveness of online training were mixed. Communicating via technology limited the ability to see or accurately read facial expressions and body language, emotions and reactions are harder to interpret (and easier to hide). Immediacy, warmth and human connection were harder to achieve. Other published studies also found mixed evidence in terms of acceptability and desirability of online training in domestic or sexual violence [34, 35].

Remote advocacy was broadly seen as acceptable and effective, and to some even desirable. Service users felt well supported by the IRIS programme throughout the first national lockdown, considering their good outcomes to be a direct reflection of the increased and responsive communication with advocates. The majority of advocates also reported positive support outcomes for their service users, although difficulties accessing onward support (creating a need to emotionally ‘hold’ service users while they waited for other support) were highlighted. This finding could be due to the precise and unusual set of circumstances brought about by the pandemic and accompanying national lockdowns rather than remote support per se; many other usual support services became (at least partially) unavailable during the first lockdown [36–38].

Strengths of this study include the multiple instruments used for data collection, allowing us to triangulate survey and quantitative referral data with interviews. Secondly, those surveyed and interviewed included relevant stakeholders involved in delivering the IRIS Programme (AEs and CLs), as well as relevant stakeholders involved as beneficiaries (clinicians and patients/service users). While the survey response rates were reasonable (39 out of 81 AEs, 25 out of 36 CLs, 115 clinicians (denominator unknown), and 18 out of 70 other members the IRIS Network), we acknowledge the likelihood of non-response bias. Self-selection may be particularly pertinent to participating clinicians. This is even more relevant if we consider that interviewees were selected from a subsample of survey respondents.

This study aimed to produce evidence around the effectiveness and acceptability of the IRIS programme in its new format. Time limitations were consequently stringent. The study was carried out in the summer 2020 – only four months into the remote delivery of IRIS advocacy and only two months into the online training delivery. This limitation meant that few online IRIS training sessions had taken place at the time. The short timeframe also limited opportunities for recruiting interviewees, particularly service users who were recruited through referral.

The findings from this research are in line with and supported by other studies regarding online and phone consultations, presentations by survivors of DA in healthcare settings, and provision of remote support [27, 36]. A report by the Health Foundation highlighted that many healthcare services had to adapt as a result of the pandemic and move towards a remote mode of working and that, in general, services that managed to adapt well relied on quickness and responsiveness as enablers of change [39]. A directly comparable qualitative study, aiming to understand the adaptations and impact of remote DVA training in IRIS-trained general practices by exploring perspectives of those delivering and receiving training [38] found benefits to widening the accessibility of training to greater numbers of busy healthcare professionals. This research reported a distinct trade-off between accessibility and levels of engagement – an anxiety shared by many of our participants.

Our finding of a short-term reduction in the number of referrals into the IRIS Programme may seem counter-intuitive in the face of increased DA reporting during lockdowns [26, 40]. Furthermore, the importance of general practice as a place for survivors to seek help during the pandemic has been reaffirmed [37]. However, our analysis is in line with findings from Murphy and colleagues, who also have observed a decline in general practice consultations in March and April 2020, and by July 2020 90% of all consultations were taking place over video or phone [41]. The reduction in consultations immediately after lockdown could partially explain the reduction in referrals to the IRIS programmes.

Remote consultations remain a common feature of general practice, suggesting they have been incorporated to current practice and will remain so in the future [42]. Preventing and responding to domestic abuse should be a strategic priority for health commissioners, and healthcare professionals are expected to be suitably trained to create safe opportunities to ask about and respond to disclosures of domestic abuse [43]. As, for the foreseeable future, a large proportion of general practice consultations will be conducted remotely it is therefore essential that clinicians are equipped to navigate concerns about domestic abuse during remote consultations. Our finding that clinicians feel less confident to address domestic abuse during remote consultations therefore has implications for policymakers and commissioners responsible for improving the healthcare response to domestic abuse; we recommend commissioning evidence-based interventions, of which the training element must improve clinicians’ confidence and skills to address DA remotely.

Future research could focus on the effectiveness of online training by comparing IRIS referrals from practices trained online to IRIS referrals from comparable practices trained face-to-face. Additionally, an analysis comparing training feedback between cohorts of clinicians trained face-to-face and online is also needed, as is a comparison of patient feedback for patients supported face-to-face compared with patients supported virtually. These self-reported outcomes, in addition to a further round of in-depth interviews, should provide meaningful insight.

## Conclusions

This rapid research found the adaptation of the IRIS Programme to remote training and support to be acceptable and desirable for AEs, clinicians and victim-survivors. While technology was mentioned as a barrier to this adaptation by all stakeholders, most clinicians felt confident addressing domestic abuse over the phone and online (although less so than face-to-face), and their victim-survivor patients felt well supported remotely. This study contributes to practice by asserting the desirability and acceptability of training clinicians to be able to identify, ask about DA and refer to the IRIS programme during telephone/online consultations. This is of relevance to health and public health commissioners when making commissioning decisions to improve the general practice response to domestic abuse.

### Electronic supplementary material

Below is the link to the electronic supplementary material.

Supplementary Material 1

## Data Availability

The datasets used and/or analysed during the current study are available from the corresponding author on reasonable request.

## Footnotes

1 In this question AEs could select multiple options from a list, thus providing a more nuanced response. Therefore the total sum is not necessarily 100%.

2 Prior to March 2020, AEs would provide the majority of their support to patients at the general practice where that patient is registered, and so became regular visitors who became embedded within the IRIS-trained practices in the local area.

## Abbreviations

**AE**: Advocate Educator - key member of a local IRIS team who delivers training and ongoing consultancy to general practice teams and supports patients affected by domestic abuse who are referred to her by general practice clinicians
**CL**: Clinical Lead – a local practicing clinician (usually a general practitioner) and a key member of a local IRIS team. The CL champions the IRIS programme amongst clinical peers and uses her/his contacts to engage practices with the IRIS programme, and co-delivers training with the AE
**DA**: Domestic abuse
**GBV**: Gender-based violence
**Host organisation**: The IRIS Partner organisation: the domestic abuse organisation that is hosting, supporting and managing the delivery of IRIS locally, including the hiring and line management of the AE(s)
**IRIS**: Identification and Referral to Improve Safety. The IRIS programme is an evidence based domestic violence and abuse training, support and referral programme developed to improve the general practice response to domestic abuse
**IRISi**: IRISi is a not-for-profit social enterprise established to improve the healthcare response to gender-based violence. IRISi works to sustain and expand the IRIS programme, and to collaborate with researchers to develop other evidence-based gender-based violence health interventions into commissionable models
**IRIS network**: The IRIS network refers to IRISi and the IRIS partners involved in delivering the IRIS service across different sites
**IRIS partners**: The host organisations delivering IRIS in their local areas
**IRIS site**: A defined geographical area in which the IRIS programme is delivered, e.g. ‘Bristol’, or ‘Hackney’
**IRIS team/local IRIS team**: The key individuals involved in delivering the IRIS programme in an IRIS site, comprising of the Advocate Educator/s and Clinical Lead/s
**Patient / service user (SU)**: Woman identified in clinical practice as a victim of DA supported by AE
**Service Manager**: The individual within the IRIS partner organisation who is responsible for managing the IRIS service in that IRIS site and for line managing the Advocate Educator/s
**VAWG**: Violence Against Women and Girls
