# Supplementary material for: COVID-19 adaptations to a training and support programme to improve primary care response to domestic abuse: a mixed methods rapid study
Source: BMC Prim Care. 2024 Jan 10;25:21. doi: 10.1186/s12875-023-02203-5 (PMC10777646; doi:10.1186/s12875-023-02203-5)
Supplement: Supplementary file 1 — Supplementary Material 1 [file 12875_2023_2203_MOESM1_ESM.docx]

# Appendix 1: Survey instruments

## Survey a: The perceived value of the IRIS Programme, the IRIS network, and IRISi work and support - for advocate educators, clinical leads, service managers and commissioners

Section 1: Introduction:

The IRIS Programme for general practices is commissioned and implemented across England, Wales and Northern Ireland. This survey uses the following definitions:

- The IRIS Programme = the copyrighted IRIS model and training package, that is (currently) commissioned in 33 areas
- IRISi = the not-for-profit social enterprise established to improve the healthcare response to gender-based violence. The IRIS Programme is IRISi’s flagship intervention. IRISi function as the national hub for the IRIS Programme and the IRIS Network; IRISi support areas to commission, implement and maintain the IRIS Programme
- IRIS sites = the local area in which the IRIS programme is commissioned.
- The IRIS Network = all of the IRIS sites (and the people involved in delivering the IRIS Programme.

IRISi are keen to understand what different IRIS stakeholders’ perceive as valuable and desirable elements of delivering the IRIS Programme and being part of the IRIS Network. This survey is to explore this topic.

1. What is your role in the IRIS programme?
   1. Advocate Educator
   2. Back up Advocate Educator
   3. Service Manager
   4. Clinical Lead
   5. Commissioner
   6. Other (please specify)
2. How long has the IRIS Programme been commissioned in your area?
   1. Less than two years
   2. Two to four years
   3. Five years or more
   4. I’m not sure

Section 2: Commissioning and delivering the IRIS Programme

1. What aspects of delivering *the IRIS Programme* are of (most) importance to you?

[please rank these aspects from 1-8, with 1 being the most important, and 8 being the least important]

[option to rank bullet points, and space for free text]

- Knowing that you are commissioning/managing/delivering an intervention that is proven to be effective
- Knowing that you are commissioning/managing/delivering an intervention that is proven to be cost effective
- Having a model that encompasses a tailored training programme, referral pathways, ongoing advice for staff at participating practices, and advocacy and support for patients [this is ‘the IRIS model’]
- Provision of readymade training resources to be used locally
- Initial training from IRISi (the IRIS Train the Trainers course)
- Ongoing support from IRISi
- Support from the IRIS network
- Other (please detail)

1. Would you like to see the continued commissioning of the IRIS Programme in your area in the medium term (e.g. the next 2 to 3 years? How about the next 5?)? Please could you explain your answer?

[free text]

*Prompts:*

- *value of the network*
- *role of IRISi support*
- *benefits/disadvantage of delivering the official IRIS Programme*
- *financial considerations*
- *Other (please detail)*

1. If your role involves any work around the *commissioning* of the IRIS Programme in your area (such as writing bids/business cases for funding), please comment on whether being part of the IRIS Network and/or having access to IRISi advice/guidance supports you in this? How could this be improved?

[free text]

Section 3: IRISi’s work

1. Which aspects of *IRISi’s work* do you value (most)?

[please rank these aspects from 1-9, with 1 being the most important, and 9 being the least important]

[option to rank bullet points, and space for free text]

*Prompts:*

- *Provision of the evidence-based IRIS model*
- *Provision of updates to training materials and guidance documents*
- *Endorsement of IRIS-related work by national bodies, such as the RCGP*
- *Initial training and support to set up IRIS in your area and recruit and train the new team*
- *Ongoing support and guidance by IRISi to deliver the IRIS programme in your area*
- *Facilitation of the IRIS Network*
- *The opportunity to feed into and add to a national voice in the DVA-health policy sector*
- *Links with academic research collaborations exploring IRIS-type models in different healthcare settings*
- *Other (please detail).*

1. What could IRISi provide that *would* be of extra value to you?

[option to select multiple bullet points, and to add free text]

*Prompts:*

- *N/A (I am happy with the work that IRISi does)*
- *More/different updates to training materials and guidance documents (please detail)*
- *More/different endorsement of IRIS-related work by national bodies, such as the RCGP (please detail)*
- *More/different initial training and support to set up IRIS in your area and recruit and train the new team (please detail)*
- *More/different ongoing support and guidance by IRISi to deliver the IRIS programme in your area (please detail)*
- *Greater/different facilitation of the IRIS Network (please detail)*
- *More/different opportunities to feed into and add to a national voice in the DVA-health policy sector (please detail)*
- *More/different links with academic research collaborations exploring IRIS-type models in different healthcare settings (please detail)*
- *Other (please detail).*

Section 4: The IRIS Network

1. Do you feel part of the IRIS Network? Please could you explain your answer?

[free text]

1. What aspects of *the IRIS network* do you value (most)?

[option to select multiple bullet points, and to add free text]

*Prompts:*

*Feeling part of a national network with a shared vision/mission to improve clinical practice and improve patients’ access to specialist support*

*Having the credibility and reputation of a national brand to support your work*

*Increased opportunities to share good practice and learning with other localities*

*Opportunity to link up with other local IRIS teams on an informal basis, for guidance/support/shadowing opportunities/sharing best practice*

*Face-to-face IRIS Network events facilitated by IRISi*

*Members area of the IRISi website (forthcoming)*

*Social media*

*Online IRIS Network events facilitated by IRISi (forthcoming)*

*Opportunity to participate in developments and improvements to the IRIS Programme*

*Other (please detail)*

1. What could the IRIS Network provide that you *would* value, that is not provided at the moment?

[option to select multiple bullet points, and to add free text]

*Prompts:*

- *N/A (I am happy with what the IRIS Network provides)*
- *More/different communications (such as an IRIS Network newsletter) (please detail)*
- *More/different IRIS events, including events for IRIS service managers or IRIS commissioners (please detail)*
- *More/different opportunities to link up with other IRIS sites (please detail)*
- *More/different opportunities to participate in developments and improvements to the IRIS programme (please detail)*
- *Other (please detail)*

Section 5: Summary

1. Please comment on your perception of the value of:
   1. The IRIS Programme [free text]
   2. IRISi’s work and support [free text]
   3. being part of the IRIS Network. [free text]
2. Please tell us anything else that is important to you about the IRIS Programme, the IRIS Network, and/or IRISi’s support and areas of work.

[free text]

Thank you for taking the time to complete this survey. We really value your opinions. We will consider everyone’s feedback, and while we will endeavour to take all your comments on board we acknowledge that we may be unable to act on every suggestion.

## Survey b: Advocate educator questions

Support for patients

1. At the outset of the COVID-19 pandemic and accompanying restrictions on face-to-face contact, did you already have a caseload of IRIS patients/clients/service users?

- Yes, full caseload
- Yes, partial caseload
- No, I had only recently started working as an AE and so didn’t yet have any cases *(skips to Q4)*

[survey branch logic to open additional question for those who had a full or partial caseload]

1. Before the COVID-19 pandemic, which of the following statements best describes the split between service users who you supported face-to-face, and service users who you supported remotely?
   1. Before COVID-19, most/nearly all my service users preferred to receive face-to-face support
   2. Before COVID-19, roughly equal numbers of my service users preferred to receive face-to-face support, and remote support
   3. Before COVID-19, most/nearly all of my service users preferred to receive remote support
   4. We are assuming that, at the outset of the pandemic, you would have offered all your service users the option to continue to receive support through remote methods (e.g. phone/video call, email, text, Whatsapp, etc). Of those service users who you were supporting at the outset of the pandemic, please use the grid below to indicate the proportions of your service users’ responses to your suggestion to transfer to remote support, and their reasons for their responses:

|  | Proportion of service users (SU) to whom this applies | | | | |
| --- | --- | --- | --- | --- | --- |
|  | All/nearly all | Most | About half | Significant minority | Tiny minority |
| Service users were happy to transfer to remote support |  |  |  |  |  |
| Service users had reservations about transferring to remote support, but did anyway |  |  |  |  |  |
| Service users were physically able to take up the offer of remote support, but chose not to |  |  |  |  |  |
| Service users were unable to transfer to remote support because they struggled to find a safe space/time to be in contact with me (e.g. living with the perpetrator) |  |  |  |  |  |
| Service users were unable to transfer to remote support because they were unable to find a private space/time to be in contact with me (e.g. living with children/other family members) |  |  |  |  |  |
| Service users were unable to transfer to remote support because they had additional caring/work responsibilities |  |  |  |  |  |
| Service users were unable to transfer to remote support because they remote support is not (easily) accessible to them (e.g. interpreter needed) |  |  |  |  |  |
| Service users were unable to transfer to remote support because they do not have access to the means for remote support (e.g. no mobile/laptop/internet access) |  |  |  |  |  |

- 1. Any additional comments/detail about whether or not service users were able to take up remote support?

[free text]

- 1. If any of your service users were reluctant to take up remote support, or were physically able to, but chose not to, what reasons did they cite for their reluctance/choice not to?

[free text]

1. Which of the following statements best describes the general trend in feedback you have received from service users patients (either verbally/informally, or on patient feedback forms), since you began providing more/only remote support:

- Service users are just as positive about the support as they normally are
- Service users are less positive about the support than they normally are
- The only service users who report that they are not finding remote support that helpful/as helpful are those who had face-to-face support before the outbreak of the pandemic
- Other (please detail)

1. The most recent IRIS quarterly data showed a significant drop in the number of IRIS referrals received, nationally, at the outset of the pandemic. How has the COVID-19 pandemic and accompanying restrictions on face-to-face contact affected the numbers of referrals you have received in your area?

- No impact (I’m still receiving the same/similar numbers of referrals)
- Initial impact (initial drop in referrals, but gradually increasing towards pre-COVID levels)
- Ongoing impact (I’m still receiving no referrals/far fewer referrals than ‘normal’)
- Other (please detail)

Since beginning to provide (more) remote support, have you noticed any change in the *type* of support that your service users want from you?

| Type of support | More | Equal | Less |
| --- | --- | --- | --- |
| Brief acknowledgement of experience (used only on its own and not in conjunction with any other type of contact or support |  |  |  |
| Emotional support |  |  |  |
| Advice and information - including housing, welfare, legal |  |  |  |
| Referral to another service in-house ie nia advocacy, FSS, Counselling |  |  |  |
| Referral to an external specialist domestic violence support service or domestic violence helpline - note the name of service |  |  |  |
| Referral to another external agency |  |  |  |
| Criminal justice intervention (police, MARAC) |  |  |  |
| Civil justice intervention (Solicitor) |  |  |  |
| Survivors' Group |  |  |  |
| Other |  |  |  |
| Inappropriate referral - no DVA |  |  |  |

1. Please describe:

[free text]

*Prompts:*

- *More emotional and mental health support?*
- *Support around new topics, e.g. anxieties around physical health/lockdown restrictions?*
- *More financial advice/support?*
- *More practical support, e.g. around safety measures, housing, etc?*
- *Greater need to ‘hold’ cases because some of the services you usually refer service users on to are not open to new referrals?*
- *Greater need to ‘hold’ cases because some/all of the informal support/coping mechanisms (e.g. friends, family, and other social occasions, exercise, hobbies, getting outside, etc) that service users usually use/access is unavailable to them*
- *Other (please detail)*

1. Has the change in the methods of support, and the types of support provided, had an impact upon you as a domestic abuse advocate?
   - Yes [branch logic to open subquestion]
   - No

If you answered yes, please describe:

[free text]

*Prompts:*

- *Have you felt able to connect with services users and build up a rapport when support is conducted remotely?*
- *Have you felt qualified/experienced enough to provide the types of support that your service users have wanted/needed?*
- *Have there been any changes to your overall caseload in terms of numbers or intensity/complexity of support?*
- *Have you had access to the tech/IT that you’ve needed to be able to support service users?*
- *Have you felt confident/that you have the skills to use the tech/IT to connect with service users?*
- *Other (please detail)*
- Have you have felt that anything has been lacking in what you have been able to offer/provide to service users? Yes [branch logic to open subquestion]
- No

If you answered ‘yes’, what would improve this for you?

[free text]

*Prompts:*

1. *Access to tech/IT e.g. Zoom/skype [free text]*
2. *Access to training on using tech/IT [free text]*
3. *More knowledge on topical subjects e.g. coronavirus, social distancing, isolation, etc [free text]*
4. *Other (please detail) [free text]*
5. Thinking about case outcomes, what impact, if any, has the change in the methods of support had for the service users you are supporting?

[free text]

*Prompts:*

- *Any change in how effective you feel the emotional support you are able to provide is for service users?*
- *Any change in how useful/comprehensive/up-to-date you feel the advice and information you are able to provide for service users is?*
- *Any change in how able you have been to support service users with the criminal justice system?*
- *Any change in how able you have been to support service users with the civil justice system?*
- *Any change in how able you have been to refer service users to other in-house services?*
- *Any change in how able you have been to refer service users to external specialist DVA/VAWG services?*
- *Any change in how able you have been to refer service users to generic external services (e.g. housing advice, financial advice, etc)?*
- *Other (please detail)*

1. Do you anticipate an influx in referrals once social distancing measures are relaxed and patients begin to attend their general practices in person again?

- Yes
- No

What could help support you to prepare for this influx?

[free text]

Support for clinicians

1. Are you still receiving enquiries and queries from clinicians at practices in your area?

- Same number as usual
- More enquiries and queries than usual
- Fewer enquiries and queries than usual
  1. Has the COVID-19 pandemic led to new questions and concerns from clinicians at your practice?
- No, same types of questions as usual
- Yes (please detail)
  1. Have you felt able to respond effectively to these?
- Yes (please detail)
- No (please detail)
  1. What support would help you to feel better able to respond?

[free text]

Questions for both AEs and CLs

Training for practices

1. IRISi have recently reworked and disseminated training materials for IRIS teams to use to deliver online training sessions for general practices in their area. Which of the following best describes the training element of the IRIS programme in your area at the moment?

- I have already delivered some training sessions online for local practices
- I have some online training sessions booked in, but they haven’t taken place yet
- I have had some requests for online training from local practices, but haven’t followed this up yet
- I haven’t approached any practices/had any discussions with practices about delivering online training sessions

1. Who will/who is delivering IRIS training in your area at the moment?

- AE and CL
- AE pairing up with another AE
- AE alone

1. Which of the following statements apply to how you are feeling about delivering IRIS training online? (Select all that apply)

- I am looking forward to delivering online training
- I have no strong feelings about delivering online training
- I am not looking forward to delivering online training
- I think clinicians will see the need for the training more than they have tended to in the past, because DA has been covered by the media much more
- I think clinicians will have more time than usual for the training, because people are avoiding using health services unless they really need to, so general practice workloads are lower than normal
- I think it will be harder to keep people engaged with a training session when it is delivered online
- I am worried about the IT/tech aspects of delivering training online
- I don’t think clinicians will come away with the same level of understanding about domestic abuse as they do from face-to-face IRIS training (please detail why)
- I think clinicians will find it harder than normal to put IRIS training into practice (please detail why)
- I don’t think training a practice online will lead to as many IRIS referrals as training a practice face-to-face normally does (please detail why)
- Other (please detail)

1. Please tell us anything else you think is important about how any other aspects of the IRIS programme are functioning at the moment.

[free text]

*Prompts:*

- *Are steering meetings happening? How often? Is this the same as usual? Are they attended by everyone who normally attends?*
- *Have you been able to promote the IRIS programme since the outset of the pandemic? How are you doing this? Has it been effective?*

1. Please tell us anything else you think is important about the effectiveness or acceptability of the IRIS programme as it is currently being delivered.

[free text]

If you would be willing to be interviewed about your views on the acceptability and effectiveness of the IRIS programme as it is currently being delivered, please add your contact details here:

## Survey c: Clinical Lead questions

Training for practices

1. IRISi have recently reworked and disseminated training materials for IRIS teams to use to deliver online training sessions for general practices in their area. Which of the following best describes the training element of the IRIS programme in your area at the moment?

- I have already delivered some training sessions online for local practices
- I have some online training sessions booked in, but they haven’t taken place yet
- I have had some requests for online training from local practices, but haven’t followed this up yet
- I haven’t approached any practices/had any discussions with practices about delivering online training sessions

1. Who will/who is delivering IRIS training in your area at the moment?

- AE and CL
- AE pairing up with another AE
- AE alone

1. Which of the following statements apply to how you are feeling about delivering IRIS training online? (Select all that apply)

- I am looking forward to delivering online training
- I have no strong feelings about delivering online training
- I am not looking forward to delivering online training
- I think clinicians will see the need for the training more than they have tended to in the past, because DA has been covered by the media much more
- I think clinicians will have more time than usual for the training, because people are avoiding using health services unless they really need to, so general practice workloads are lower than normal
- I think it will be harder to keep people engaged with a training session when it is delivered online
- I am worried about the IT/tech aspects of delivering training online
- I don’t think clinicians will come away with the same level of understanding about domestic abuse as they do from face-to-face IRIS training (please detail why)
- I think clinicians will find it harder than normal to put IRIS training into practice (please detail why)
- I don’t think training a practice online will lead to as many IRIS referrals as training a practice face-to-face normally does (please detail why)
- Other (please detail)

1. Please tell us anything else you think is important about how any other aspects of the IRIS programme are functioning at the moment.

[free text]

*Prompts:*

- *Are steering meetings happening? How often? Is this the same as usual? Are they attended by everyone who normally attends?*
- *Have you been able to promote the IRIS programme since the outset of the pandemic? How are you doing this? Has it been effective?*

1. Please tell us anything else you think is important about the effectiveness or acceptability of the IRIS programme as it is currently being delivered.

[free text]

1. How confident do you feel to identify and ask about domestic abuse (DA) during telephone/video consultations?
   - Very confident
   - Somewhat confident
   - Neither confident nor unconfident
   - Somewhat unconfident
   - Very unconfident
   1. How does this compare with your level of confidence to identify and ask about domestic abuse during face-to-face consultations?
   - I am much more confident identifying and asking about DA during face-to-face consultations
   - I am somewhat more confident identifying and asking about DA during face-to-face consultations
   - I have the same level of confidence in identifying and asking about DA for both face-to-face consultations and telephone/video consultations
   - I am somewhat less confident identifying and asking about DA during face-to-face consultations
   - I am much less confident identifying and asking about DA during face-to-face consultations
   1. Please explain your answer

[free text]

*Prompts/you might want to consider/include the following in your answer:*

- *Ensuring confidentiality of the consultation*
- *Ensuring safety of patient before asking about DA*
- *Your opportunities for observing symptoms/conditions associated with DA*
- *Your opportunities to read a patient’s body language/demeanour*
- *Whether in your experience patients find it easier to talk about difficult things when they are face-to-face, or when you can’t see their face*
- *Ease/difficulty of accessing interpreters, where needed, in face-to-face/remote consultations*
- *Other (please describe)*

1. How confident do you feel to respond to disclosures of domestic abuse (DA) during telephone/video consultations (including validating the patient’s experience, assessing the risk, offering a referral, and recording the disclosure)?
   - Very confident
   - Somewhat confident
   - Neither confident nor unconfident
   - Somewhat unconfident
   - Very unconfident
2. How does this compare with your level of confidence to respond during face-to-face consultations?
   - I am much more confident responding during face-to-face consultations
   - I am somewhat more confident responding during face-to-face consultations
   - I have the same level of confidence in responding for both face-to-face consultations and telephone/video consultations
   - I am somewhat less confident responding during face-to-face consultations
   - I am much less confident responding during face-to-face consultations
3. Please explain your answer

[free text]

*Prompts/you might want to consider/include the following in your answer:*

- *Gauging the patient’s response with/without body language/facial expressions etc*
- *Conveying your validating, supportive response with/without body language/facial expressions etc*
- *Whether you have the same/more/less knowledge as to which DA services are open for referrals at the moment*
- *Whether you have the same/more/less knowledge as to the support that your practice’s IRIS Advocate Educator can provide at the moment*
- *Other (please describe)*

1. In your clinical practice, have you asked patient/s about domestic abuse during:
   - Face-to-face consultations
     - Yes
     - No
   - Phone/video consultations
     - Yes
     - No
2. In your clinical practice, have you responded to disclosures of domestic abuse (whether prompted by your questions, or unprompted) during:
   - Face-to-face consultations
     - Yes
     - No
   - Phone/video consultations
     - Yes
     - No
3. In your clinical practice, have you referred patient/s to your practice’s IRIS Advocate Educator during:
   - Face-to-face consultations
     - Yes
     - No
   - Phone/video consultations
     - Yes
     - No
4. How valuable/important is it to you that your practice is part of the local IRIS Programme?
   - Very valuable/important
   - Quite valuable/important
   - Not valuable/important
5. Please explain your answer

[free text]

*Prompts/you might want to consider/include the following in your answer:*

- *Whether or not the IRIS training is relevant/interesting/helpful*
- *Whether or not written guidance documents produced/disseminated by/through the IRIS programme are relevant/interesting/helpful*
- *Whether or not the access to a named advocate (your IRIS Advocate Educator) to whom you can refer patients is helpful*
- *Whether or not your patients have reported any positive outcomes/feedback after having received support from the IRIS Advocate Educator*
- *Whether or not the access to a DA specialist (your IRIS Advocate Educator) who can give guidance and support on any questions or concerns you have about DA is helpful*
- *Other (please detail)*

1. Has your view of the value/relevance of the IRIS programme increased or decreased since the COVID-19 pandemic and accompanying restrictions led to the IRIS programme being delivered online?

- I see the IRIS programme as much more valuable/relevant now
- I see the IRIS programme as a bit more valuable/relevant now
- I think the IRIS programme has the same value/relevance now as always
- I think the IRIS programme is a bit less valuable/relevant now
- I think the IRIS programme is much less valuable/relevant now

Please explain your answer

[free text]

*Prompts/you might want to consider/include the following in your answer:*

- - - I am more aware of DA now as it has been in the media more often
    - I have more time now to engage with training than in the past
    - The IRIS programme has been able to provide support to patients who otherwise would not have been able to access any support during lockdown
    - I preferred the training when it was face-to-face
    - My patients were more willing to access advocacy and support when it was face-to-face
    - My patients reported better outcomes when they were able to access advocacy and support face-to-face

If you would be willing to be interviewed about your views on the acceptability and effectiveness of the IRIS programme as it is currently being delivered, please add your contact details here:

## Survey d: clinician questions

1. Have you completed any IRIS training?
   - None
   - One training session
     - Face-to-face (pre COVID-19)
     - Online (post COVID-19)
   - At least two training sessions
     - All face-to-face (pre COVID-19)
     - All online (post COVID-19)
     - Mix between face-to-face (pre COVID-19) and online (post COVID-19)

*[survey form to use branch logic to open up a new question for those who have attended an online training session]*

1. We’re keen to know what you thought of the online training

*Prompts*: Sub-questions:

- *Relevance of the content covered during the sessions*
  - Likert scale: Very relevant, somewhat relevant, neither relevant nor irrelevant, somewhat irrelevant, very irrelevant
- *What about the methods/activities used during the training? Were these sufficiently engaging/interactive?*
  - Very engaging, quite engaging, neither engaging nor disengaging, somewhat disengaging, very disengaging
- *Did you have a safe, quiet space from which to join the training?*
  - Yes/no (if no, please detail reason/s)
- *Did you have any distractions that you had to manage (e.g. children, other family members)?*
  - No/yes (if yes, please detail reason/s)
- *Did you have access to a device that allowed you to use the full functionality of the online training?*
  - Yes/no (if no, please detail reason/s)
- *Did you have any technical difficulties in connecting to the training session?*
  - No/yes (if yes, please detail reason/s)
- *Did you find it straightforward to access related resources and materials before/after the training session*
  - Yes/no (if no, please detail reason/s)
- *Fitting training into the working day, e.g. was this easier or harder with the online training?*
  - Much easier, somewhat easier, neither easier nor harder, somewhat harder, much harder
- *DA can be a difficult subject to talk about. Was it easier or harder to attend DA training when you weren’t in the same physical space as the trainer/s and/or your colleagues?*
  - Much easier, somewhat easier, neither easier nor harder, somewhat harder, much harder
- *Other (please explain)*
- *Is there anything else about the online training that you would like to add? [free text]*

*[survey form to use branch logic to open up a new question for those who have attended* ***both*** *a face-to-face training session and an online training session]*

1. How did your experiences of face-to-face and online IRIS training compare with each other?
2. Did you find one type of training more effective than the other? Why? [free text]
3. Did you learn more from one type of training? Why? [free text]
4. Do you find one type of training more enjoyable/engaging than the other? Why? [free text]
5. Did one type of training have more of an impact on your clinical practice than the other? Why? [free text]
6. How confident do you feel to identify and ask about domestic abuse (DA) during telephone/video consultations?
   - Very confident
   - Somewhat confident
   - Neither confident nor unconfident
   - Somewhat unconfident
   - Very unconfident
   1. How does this compare with your level of confidence to identify and ask about domestic abuse during face-to-face consultations?
   - I am much more confident identifying and asking about DA during face-to-face consultations
   - I am somewhat more confident identifying and asking about DA during face-to-face consultations
   - I have the same level of confidence in identifying and asking about DA for both face-to-face consultations and telephone/video consultations
   - I am somewhat less confident identifying and asking about DA during face-to-face consultations
   - I am much less confident identifying and asking about DA during face-to-face consultations
   1. Please explain your answer

[free text]

*Prompts/you might want to consider/include the following in your answer:*

- *Ensuring confidentiality of the consultation*
- *Ensuring safety of patient before asking about DA*
- *Your opportunities for observing symptoms/conditions associated with DA*
- *Your opportunities to read a patient’s body language/demeanour*
- *Whether in your experience patients find it easier to talk about difficult things when they are face-to-face, or when you can’t see their face*
- *Ease/difficulty of accessing interpreters, where needed, in face-to-face/remote consultations*
- *Other (please describe)*

1. How confident do you feel to respond to disclosures of domestic abuse (DA) during telephone/video consultations (including validating the patient’s experience, assessing the risk, offering a referral, and recording the disclosure)?
   - Very confident
   - Somewhat confident
   - Neither confident nor unconfident
   - Somewhat unconfident
   - Very unconfident
2. How does this compare with your level of confidence to respond during face-to-face consultations?
   - I am much more confident responding during face-to-face consultations
   - I am somewhat more confident responding during face-to-face consultations
   - I have the same level of confidence in responding for both face-to-face consultations and telephone/video consultations
   - I am somewhat less confident responding during face-to-face consultations
   - I am much less confident responding during face-to-face consultations
3. Please explain your answer

[free text]

*Prompts/you might want to consider/include the following in your answer:*

- *Gauging the patient’s response with/without body language/facial expressions etc*
- *Conveying your validating, supportive response with/without body language/facial expressions etc*
- *Whether you have the same/more/less knowledge as to which DA services are open for referrals at the moment*
- *Whether you have the same/more/less knowledge as to the support that your practice’s IRIS Advocate Educator can provide at the moment*
- *Other (please describe)*

1. In your clinical practice, have you asked patient/s about domestic abuse during:
   - Face-to-face consultations
     - Yes
     - No
   - Phone/video consultations
     - Yes
     - No
2. In your clinical practice, have you responded to disclosures of domestic abuse (whether prompted by your questions, or unprompted) during:
   - Face-to-face consultations
     - Yes
     - No
   - Phone/video consultations
     - Yes
     - No
3. In your clinical practice, have you referred patient/s to your practice’s IRIS Advocate Educator during:
   - Face-to-face consultations
     - Yes
     - No
   - Phone/video consultations
     - Yes
     - No
4. How valuable/important is it to you that your practice is part of the local IRIS Programme?
   - Very valuable/important
   - Quite valuable/important
   - Not valuable/important
5. Please explain your answer

[free text]

*Prompts/you might want to consider/include the following in your answer:*

- *Whether or not the IRIS training is relevant/interesting/helpful*
- *Whether or not written guidance documents produced/disseminated by/through the IRIS programme are relevant/interesting/helpful*
- *Whether or not the access to a named advocate (your IRIS Advocate Educator) to whom you can refer patients is helpful*
- *Whether or not your patients have reported any positive outcomes/feedback after having received support from the IRIS Advocate Educator*
- *Whether or not the access to a DA specialist (your IRIS Advocate Educator) who can give guidance and support on any questions or concerns you have about DA is helpful*
- *Other (please detail)*

1. Has your view of the value/relevance of the IRIS programme increased or decreased since the COVID-19 pandemic and accompanying restrictions led to the IRIS programme being delivered online?

- I see the IRIS programme as much more valuable/relevant now
- I see the IRIS programme as a bit more valuable/relevant now
- I think the IRIS programme has the same value/relevance now as always
- I think the IRIS programme is a bit less valuable/relevant now
- I think the IRIS programme is much less valuable/relevant now

Please explain your answer

[free text]

*Prompts/you might want to consider/include the following in your answer:*

- - - I am more aware of DA now as it has been in the media more often
    - I have more time now to engage with training than in the past
    - The IRIS programme has been able to provide support to patients who otherwise would not have been able to access any support during lockdown
    - I preferred the training when it was face-to-face
    - My patients were more willing to access advocacy and support when it was face-to-face
    - My patients reported better outcomes when they were able to access advocacy and support face-to-face

If you would be willing to be interviewed about your views on the acceptability and effectiveness of the IRIS programme as it is currently being delivered, please add your contact details here:

_________

# Appendix 2: Participant information sheets

## Participant Information Sheet for IRIS Advocate Educators, IRIS Clinical Leads, IRIS-trained clinicians, and IRISi Regional Managers

Study title: Rapid research to understand the effectiveness and acceptability of remote delivery of the IRIS programme

*We would like to invite you to take part in our research study to understand the efficacy and acceptability of remote delivery of the IRIS programme. Before you decide, please read this information so that you understand why the research is being done and what it involves. Please ask if anything is unclear.*

**What is the purpose of the study?**

IRIS is a specialist domestic violence and abuse (DVA) training, support and referral programme for General Practices that has been positively evaluated in a randomised controlled trial. IRIS is a collaboration between primary care and third sector organisations specialising in DVA.  Core areas of the programme include ongoing training, education and consultancy for the clinical team and administrative staff, care pathways for primary health care practitioners and an enhanced referral pathway to specialist domestic violence services for patients with experience of DVA.

Until March 2020 the vast majority of IRIS-related work had always taken place face-to-face. Since the outset of the COVID-19 pandemic and accompanying restrictions on face-to-face contact, nearly every aspect of the IRIS programme has shifted to being delivered remotely.

The purpose of this study is to begin to build a picture of the effectiveness and acceptability of the IRIS programme when it is delivered remotely.

This study is being undertaken by the rapid research team at IRISi. IRISi is a not-for-profit social enterprise established to improve the healthcare response to gender-based violence. The IRIS programme is IRISi’s flagship programme. IRISi supports areas to commission, implement and maintain their local IRIS programme.

**Why have I been invited?**

You are an IRIS Advocate Educator/IRIS Clinical Lead/IRIS-trained clinician/ IRISi Regional Manager. Your experience and opinions will help us to understand whether remote delivery of the IRIS programme is effective and acceptable and will enable us to improve and adapt the IRIS programme for the post-COVID-19 health sector.

**Do I have to take part?**

No, it is up to you to decide whether to participate in the study. If you agree to take part, we will seek your consent. You are free to withdraw from the study at any time without giving us a reason.

**What will happen to me if I take part and what will I have to do?**

- For IRIS Advocate Educators, IRIS Clinical Leads, and IRIS-trained clinicians:

There are two aspects to the study. We would ideally like participants to complete an online questionnaire and a semi-structured interview conducted by phone/video call, but you may choose to participate in only one of these.

- For IRISi Regional Managers:

We would like participants to take part in a semi-structured interview conducted by phone/video call.

1. *Questionnaire:* we will ask you to complete a short, computerised questionnaire. For Advocate Educators and Clinical Leads, the questionnaire will ask you about your experiences of and views on delivering the IRIS programme in your area since the programme shifted to remote delivery, i.e. online IRIS training sessions and remote advocacy. For IRIS-trained clinicians, the questionnaire will ask you about your experiences of and views on attending IRIS training and your perception of the value of the IRIS programme. Individual level identifiable data (such as your email address) will be seen by the rapid research team at IRISi (the Data Scientist and the Development Lead for Social Franchising). Individual questionnaire responses will not be shared with the wider IRISi team; anonymous collated data will be shared with relevant IRISi team members to enable planning for improvements to the IRIS programme. Completion of the questionnaire will be taken as implied consent. Data will be anonymised and aggregated for publication and reports so that it is not identifiable.
2. *Semi-structured interviews*: participants will be invited to take part in one interview. A researcher will interview you on your own in a confidential place. This could be over the phone or via video call. For each participant type, the interviewer will be the following:

| **Participant** | **Interviewer** |
| --- | --- |
| Advocate Educators, and  Clinical Leads, and  IRIS-trained clinicians | IRISi Data Scientist, or  IRISi Development Lead for Social Franchising, or  one of the IRISi Regional Managers |
| IRISi Regional Managers | IRISi Data Scientist |

You will be sent a consent form before the interview and can either sign this electronically, or provide verbal consent. During the interview, you will be asked about your experience and views on the effectiveness and acceptability of remote delivery of the IRIS programme. There are no right or wrong answers, we are interested in your views and opinions. The interview will last about 45 minutes and will be recorded on a password protected digital recorder.

**What are the possible disadvantages and risks of taking part?**

It is unlikely that your participation in this study will cause you harm. You may discuss topics during the interview that feel difficult, including working relationships, and you can choose what to share with the researcher. You can stop the interview at any point and do not have to answer any questions that you are not comfortable with.

**What are the possible benefits of taking part?**

We cannot promise that you will receive direct benefits from the study. However, findings from the questionnaires and interviews will be used to plan improvements to the IRIS programme, from which you may benefit.

**What will happen if I don’t want to carry on with the study?**

If you decide to take part, but then later change your mind that is okay. If you change your mind during an interview then we will ask you if we can keep or delete the recording that we have already made. If you change your mind after you have completed a questionnaire or interview then we will delete your data (questionnaire, audio recording and transcript) and not use it in our analysis. You can ask for your data to be removed from the study up to one week after you have completed a questionnaire or interview.

**Will my taking part in this study be kept confidential?**

Identifiable data collected using the questionnaire will be seen by the IRISi rapid research team. Anonymised and collated data may be shared with wider IRISi team members to help us improve the IRIS programme. Only anonymised and aggregated data will be published or disseminated.

For semi-structured interviews, only the rapid research team (Data Scientist, Development Lead, and the Regional Manager who conducted the interview) will know who participated in an interview and only the Data Scientist will have access to the audio recordings and transcripts. Interviews will be transcribed by an external transcription company approved by the Data Scientist which has a confidentiality agreement in place and is fully GDPR compliant. Transcripts of semi-structured interviews will be cleaned and anonymised by the Data Scientist, with removal of personal identifiers such as names, country and place names and clinician names. Transcripts will be analysed by the Data Scientist and the Development Lead. We will take steps to ensure anonymity when reporting quotes in publications or other communication of our findings.

Despite every effort taken to maintain the anonymity of all research participants, there does remain a possibility that you will be identifiable by the nature of your responses/comments, especially to other members of the IRISi team, given the small number of participants, particularly if you are a Regional Manager for IRISi.

All of the information that we collect will be held securely by IRISi and will only be seen in full by the Data Scientist. Storage of all data will comply with the Data Protection Act and IRISi’s data protection policies. Data will be stored on password-protected computers for 3 years or when the study is finished, whichever occurs sooner. After this period electronic audio recordings will be deleted, and consent forms will be disposed of through a confidential waste service.

**What will happen to the results of the research study?**

Only the Data Scientist and the Development Lead will have access to your personal data. Anonymised findings from the study will be published in a written report for that will be used internally by IRISi, and will be shared with external parties too. We may also present our findings at conferences or through other media, and/or in a peer-reviewed journal. You will not be identified in any report, publication or conference. If you would like to receive a copy of the summary findings, please let the researcher know and provide an email address to which this can be forwarded.

**Who is organising and funding the research?**

This study is organised by IRISi using a portion of the funds received from the Health Foundation as part of the Health Foundation’s *Exploring Social Franchising* programme.

**Who has reviewed the study?**

The study has been reviewed and approved by the CEO of IRISi.

IRISi will be using information from you in order to undertake this study and will act as the data controller for this study. This means that we are responsible for looking after your information and using it properly. IRISi will keep identifiable information about you until the study is finished.

Your rights to access, change or move your information are limited, as we need to manage your information in specific ways in order for the research to be reliable and accurate. If you withdraw from the study, we will keep the information about you that we have already obtained. To safeguard your rights, we will use the minimum personally-identifiable information possible.

**Further information and contact details**

If you have any concerns about any part of the study, or would like more information, please contact the Data Scientist, Estela Barbosa on [Estela.barbosa@irisi.org](mailto:Estela.barbosa@irisi.org).

If you wish to make a complaint, please contact:

- CEO of IRISi, Medina Johnson, on [Medina.johnson@irisi.org](mailto:Medina.johnson@irisi.org).

**Thank you for taking the time to read this information leaflet**

## Participant Information Sheet for Service Users

Study title: Rapid research to understand the effectiveness and acceptability of remote delivery of the IRIS programme

*We would like to invite you to take part in our research study to understand the efficacy and acceptability of remote delivery of the IRIS programme. Before you decide, please read this information so that you understand why the research is being done and what it involves. Please ask if anything is unclear.*

**What is the purpose of the study?**

IRIS is a specialist domestic violence and abuse (DVA) training, support and referral programme for General Practices that has been positively evaluated in a randomised controlled trial. IRIS is a collaboration between primary care and third sector organisations specialising in DVA.  Core areas of the programme include ongoing training, education and consultancy for the clinical team and administrative staff, care pathways for primary health care practitioners and an enhanced referral pathway to specialist domestic violence services for patients with experience of DVA.

Until March 2020 the vast majority of IRIS-related work had always taken place face-to-face. Since the outset of the COVID-19 pandemic and accompanying restrictions on face-to-face contact, nearly every aspect of the IRIS programme has shifted to being delivered remotely.

The purpose of this study is to begin to build a picture of the effectiveness and acceptability of the IRIS programme when it is delivered remotely.

This study is being undertaken by the rapid research team at IRISi. IRISi is a not-for-profit social enterprise established to improve the healthcare response to gender-based violence. The IRIS programme is IRISi’s flagship programme. IRISi supports areas to commission, implement and maintain their local IRIS programme.

**Why have I been invited?**

You are someone who has been referred into the IRIS service. Your experience and opinions will help us to understand whether remote delivery of the IRIS programme is effective and acceptable and will enable us to improve and adapt the IRIS programme for the post-COVID-19 health sector.

**Do I have to take part?**

No, it is up to you to decide whether to participate in the study. If you agree to take part, we will seek your consent. You are free to withdraw from the study at any time without giving us a reason.

**What will happen to me if I take part and what will I have to do?**

We would like participants to take part in a semi-structured interview conducted by phone/video call.

*Semi-structured interviews*: participants will be invited to take part in one interview. A researcher will interview you on your own in a confidential place. This could be over the phone or via video call. The researcher will be the member of the IRISi team who is the national contact (the Regional Manager) for the local IRIS team in the area in which you live.

You will be sent a consent form before the interview and can either sign this electronically, or provide verbal consent. During the interview, you will be asked about your experience and views on the effectiveness and acceptability of remote delivery of the IRIS programme. There are no right or wrong answers, we are interested in your views and opinions. The interview will last about 45 minutes and will be recorded on a password protected digital recorder.

**What are the possible disadvantages and risks of taking part?**

It is unlikely that your participation in this study will cause you harm. Your IRIS Advocate Educator and the IRISi Regional Manager will take all possible steps to ensure that if you wish to take part, you can do so safely. You may discuss topics during the interview that feel difficult, and you can choose what to share with the researcher. You can stop the interview at any point and do not have to answer any questions that you are not comfortable with.

**What are the possible benefits of taking part?**

We cannot promise that you will receive direct benefits from the study. However, findings from the questionnaires and interviews will be used to plan improvements to the IRIS programme, from which you, and/or other IRIS service users, may benefit.

**What will happen if I don’t want to carry on with the study?**

If you decide to take part, but then later change your mind that is okay. If you change your mind during an interview then we will ask you if we can keep or delete the recording that we have already made. If you change your mind after you have completed a questionnaire or interview then we will delete your data (questionnaire, audio recording and transcript) and not use it in our analysis. You can ask for your data to be removed from the study up to one week after you have completed a questionnaire or interview.

**Will my taking part in this study be kept confidential?**

Identifiable data (such as your name and contact number) will be seen by the IRISi rapid research team. Anonymised and collated data may be shared with wider IRISi team members to help us improve the IRIS programme. Only anonymised and aggregated data will be published or disseminated.

For semi-structured interviews, only the rapid research team (Data Scientist, Development Lead, and the Regional Manager who conducted the interview) will know who participated in an interview and only the Data Scientist will have access to the audio recordings and transcripts. Interviews will be transcribed by an external transcription company approved by the Data Scientist which has a confidentiality agreement in place and is fully GDPR compliant. Transcripts of semi-structured interviews will be cleaned and anonymised by the Data Scientist, with removal of personal identifiers such as names, country and place names and clinician names. Transcripts will be analysed by the Data Scientist and the Development Lead. We will take steps to ensure anonymity when reporting quotes in publications or other communication of our findings.

All of the information that we collect will be held securely by IRISi and will only be seen in full by the Data Scientist. Storage of all data will comply with the Data Protection Act and IRISi’s data protection policies. Data will be stored on password-protected computers for 3 years or when the study is finished, whichever occurs sooner. After this period electronic audio recordings will be deleted, and consent forms will be disposed of through a confidential waste service.

**What will happen to the results of the research study?**

Only the Data Scientist and the Development Lead will have access to your personal data. Anonymised findings from the study will be published in a written report for that will be used internally by IRISi, and will be shared with external parties too. We may also present our findings at conferences or through other media, and/or in a peer-reviewed journal. You will not be identified in any report, publication or conference. If you would like to receive a copy of the summary findings, please let the researcher know and provide an email address to which this can be forwarded.

**Who is organising and funding the research?**

This study is organised by IRISi using a portion of the funds received from the Health Foundation as part of the Health Foundation’s *Exploring Social Franchising* programme.

**Who has reviewed the study?**

The study has been reviewed and approved by the CEO of IRISi.

IRISi will be using information from you in order to undertake this study and will act as the data controller for this study. This means that we are responsible for looking after your information and using it properly. IRISi will keep identifiable information about you until the study is finished.

Your rights to access, change or move your information are limited, as we need to manage your information in specific ways in order for the research to be reliable and accurate. If you withdraw from the study, we will keep the information about you that we have already obtained. To safeguard your rights, we will use the minimum personally-identifiable information possible.

**Further information and contact details**

If you have any concerns about any part of the study, or would like more information, please contact the Data Scientist, Estela Barbosa on [Estela.barbosa@irisi.org](mailto:Estela.barbosa@irisi.org).

If you wish to make a complaint, please contact:

- CEO of IRISi, Medina Johnson, on [Medina.johnson@irisi.org](mailto:Medina.johnson@irisi.org).

**Thank you for taking the time to read this information leaflet**

# Appendix 3: Interview topic guides

## Topic guide a: Advocate educators and clinical leads

Aims:

Understanding in more depth:

- Whether AEs and CLs find the new mode of delivery acceptable and effective

Draft guide:

Thank you for agreeing to take part in this interview. This interview aims to explore in more depth some of the content covered in the survey you have already filled in. If you recall, the survey asked you questions around the change from face-to-face training and support to remote training and support, the value perceived of face-to-face / remote support, and whether you felt IRIS training online was as effective and desirable as face-to-face.

By agreeing to be interviewed, you are effectively providing consent. Are you happy to be interviewed?

I would like to record this interview so it can be transcribed and analysed. I will be using password protected recorder and only the research team will have access to the interview audio and transcript. Are you happy for this interview to be recorded?

1. May I start by asking, what your job role is?
2. How long have you been working with the IRIS programme?

If the answer to question 1 was Clinical Lead, skip to question 9 and use Clinician Topic Guide after question 11.

1. In your experience, before the pandemic, was there a preference among supported women in terms of face-to-face support or remote support? Can you speculate the reasons for this?
2. How has this changed since the lockdown?
3. Do you feel you can support women as well as you could before, considering the circumstances?
4. What impact has the change in the methods of support, and the types of support provided, had upon you as a domestic abuse advocate?
5. Do you think this new form of support has an impact in your case outcomes? How so?
6. Has COVID-19 changed the type of support you provide for clinicians? Can you please expand?
7. Since the lockdown, IRIS training has moved online. Have you delivered any online IRIS training? How did you find it (them)?
8. (If applicable)

What would you say is the main difference between face-to-face training and online training? Can you tell me how you feel this difference could be minimised, if desirable to minimise the difference?

1. Is there anything you think I should be asking you, but haven’t asked?

Thank you for participating on this interview. I really appreciate your time and your answers are really helpful.

## Topic guide b: Clinicians

Aims:

Understanding in more depth:

- How clinicians feel in terms of discussing domestic abuse during phone/video consultations;
- What attributes of the IRIS programme they value;
- If they find online IRIS training as effective and desirable as face-to-face IRIS training.

Draft guide:

Thank you for agreeing to take part in this interview. This interview aims to explore more in-depth some of the content covered in the survey you have already filled in. If you recall, the survey asked you questions around your confidence in discussing domestic abuse over the phone or in video consultations, the value of the IRIS programme, and whether you felt IRIS training online was as effective and desirable as face-to-face.

By agreeing to be interviewed, you are effectively providing consent. Are you happy to be interviewed?

I would like to record this interview so it can be transcribed and analysed. I will be using password protected recorder and only the research team will have access to the interview audio and transcript. Are you happy for this interview to be recorded?

1. May I start by asking, what your job role is?
2. How long have you known the IRIS programme?
3. Have you attended face-to-face IRIS training? If yes, how did you find it (them)?
4. Since the lockdown, IRIS training has moved online. Have you attended any online IRIS training? How did you find it (them)?
5. (If applicable)

What would you say is the main difference between face-to-face training and online training? Can you tell me how you feel this difference could be minimised (if minimising the difference is desirable)?

1. In your opinion, why is the IRIS programme effective? Can you please expand on that?
2. In your opinion, what is the added value of the IRIS programme for your clinical practice? How about your patients? And the practice as a whole?
3. How confident do you feel to identify and ask about domestic abuse (DA)?
4. Is there a difference in your confidence level when you are doing face-to-face or online/phone consultations? Can you please expand?
5. How confident do you feel to respond to disclosures of domestic abuse (DA)?
6. Is there a difference in your confidence level when dealing with disclosures face-to-face or online/ via phone? Can you please expand?
7. Do you think the COVID-19 pandemic has changed the relevance of the IRIS programme? How so?
8. Is there anything you think I should be asking you, but haven’t asked?

Thank you for participating on this interview. I really appreciate your time and your answers are really helpful.

## Topic guide c: Service Users

Aims:

Understanding in more depth:

Do service users find phone/online IRIS advocacy (as) effective and desirable (as face-to-face IRIS advocacy?

Draft guide:

Thank you for agreeing to take part in this interview. This interview aims to explore your experiences of receiving phone/online IRIS advocacy and support, in particular whether you have found this support to be (as) effective and desirable (as face-to-face support).

By agreeing to be interviewed, you are effectively providing consent. Are you happy to be interviewed?

I would like to record this interview so it can be transcribed and analysed. I will be using password protected recorder and only the research team will have access to the interview audio and transcript. Are you happy for this interview to be recorded?

For all service users:

1. May I start by asking, roughly when were you referred to the IRIS advocate educator? Was it before or after lockdown?
2. Did you want or expect your GP/clinician to ask you about domestic abuse? If you have answered ‘yes’, please can you explain why?
3. Please can you tell me about your experience of telling your GP/clinician about the abuse you are/were experiencing?

*Prompts –*

- *What type of consultation – phone/video/face-to-face?*
- *Did the GP/clinician ask you directly about domestic abuse?*
  *Or did you disclose without being prompted?*

1. Please can you tell me about your experience of your GP/clinician’s response, when you told them what you were/are experiencing?

*Prompts –*

- *Did you feel listened to?*
- *Did you feel supported?*
- *Did your GP/clinician offer you a referral?*

For service users referred since lockdown:

1. Under the current circumstances of the COVID-19 pandemic and lockdown restrictions, is there any way that your general practice’s response could have been improved?
2. Since you were referred, how have you kept in touch with your IRIS advocate educator?
3. How easy or difficult has it been to find safe opportunities to keep in touch with your IRIS advocate educator?
4. What has your IRIS advocate educator been able to support and help you with? How do you feel about this?
5. Are there things that your IRIS advocate educator hasn’t been able to help and support you with? Is this/are these because of the pandemic and the lockdown restrictions? How do you feel about this?
6. Under the current circumstances, is there any way that you think the support provided by the IRIS service could be improved?
7. Is there anything else you think I should be asking you, but haven’t asked?

Thank you for participating on this interview. I really appreciate your time and your answers are really helpful.

For service users referred before lockdown:

1. Before lockdown, did you meet with your IRIS advocate educator face-to-face?
2. When your IRIS advocate educator explained that she would no longer be able to meet you face-to-face, how did you feel about this? Why? Do you feel differently about this now?
3. Since the beginning of lockdown, how have you kept in touch with your IRIS advocate educator?
4. How easy or difficult has it been to find safe opportunities to keep in touch with your IRIS advocate educator?
5. What has your IRIS advocate educator been able to support and help you with? How do you feel about this?
6. Are there things that your IRIS advocate educator hasn’t been able to help and support you with? Is this/are these because of the pandemic and the lockdown restrictions? How do you feel about this?
7. Under the current circumstances, is there any way that you think the support provided by the IRIS service could be improved?
8. Is there anything else you think I should be asking you, but haven’t asked?

Thank you for participating on this interview. I really appreciate your time and your answers are really helpful.

# Appendix 4: Framework analysis matrices

## Matrix a. Framework analysis of interviews – Advocate educators

| Barriers  *Advocacy:*  Service user’s restrictions at home (perpetrator more often present in house  Perpetrator using lockdown as a control/isolation tactic);  Service user’s mental ill health;  More time consuming  Other organisations (i.e. solicitors, social care) being slow to respond;    *Engaging with practices and delivering training:*  More difficult to get evaluation forms completed;  Difficult to get (extra) time commitment from practice staff (communications with practices via email are easier for them to ignore);  Technological difficulties – platforms not always working, difficulties dealing with chat while delivering training;  Online training is more intense and requires more alertness; | Enablers  *Advocacy:*  Adapting work and ‘offer’ alongside other organisations adapting;  New ways of working – text and WhatsApp messages;  AEs more accessible    *Engaging with practices and delivering training:*  More accessible for attendees;  Seeing the AE ‘live’ during training - ‘Live’ nature of online delivery is crucial to building relationships;  Additional awareness raising sessions;  Success breeds success – clinicians (and patients) having a positive perception of the IRIS service increases the usage of the IRIS service;  Flexibility around training times / to edit/adapt training materials;  Online training enables dispersed clinicians to attend;  *General*  Support from CCG (clinical commissioning group) contact promoting IRIS programme via bulletins;  Support from IRISi; |
| --- | --- |
| Losses  *Advocacy:*  Lessened ability to be present to accompany service user to appointments;  Lessened ability to deal with multiple queries in one go;  Less ‘value’ from AE time;  Not seeing body language, hearing tone of voice;  *Engaging with practices and delivering training:*  Ability to build relationships/network diminished, esp. if remote delivery is long term;  Pre-recorded training would remove personal relationship;  Informal opportunities for relationship building diminished;  Minority of practices have refused online training in preference of waiting for face to face. | Benefits  *Advocacy:*  Increased referrals  New package of advocacy support specific to pandemic  Good case outcomes  ‘no waste of time’ – increased productivity, measured quantifiably (advocacy and training)    *Engaging with practices and delivering training:*  Easier to gather practice staff for training at same time;  Practices have requested online training. |

## Matrix b. Framework analysis of interviews – Clinical leads

| Barriers  Set ways in clinical practice;  Engagement and attention of clinicians during online training (and its reflexion on referrals);  Confusion about referrals;  Uncertain whether patient is alone for a phone consultation;  Not necessarily knowing the patient (if it’s a telephone consultation);  Backlog of training;  Technical difficulties with technology -different organisations use different platforms and it’s not always easy to adapt;  Sharing views and perceptions with other members of the team;  Lack of confidence from GPs in dealing with/responding to DA remotely. | Enablers  Quick responsiveness from AEs to referrals;  Regular check-ins with the audience during training;  Practices being pro-active and putting info on their website;  Practices more interested in getting IRIS training;  Getting professional training to use online platforms;  COVID-19 guidance on telephone consultations;  IRISi online training - made it look smooth and possible;  Online training demonstrates role play, video and telephone consultations  High quality materials developed for online training and support;  Good connection between AEs and practices;  Creative response from clinicians. |
| --- | --- |
| Losses  Not seeing body language, hearing tone of voice;  Ability to hold space for people struggling with training;  Attending training alone from your home – loss of opportunity to share experience, thoughts, feelings with other trainees;  Ability to see patients at the GP practice (temporary). | Benefits  Help women responding to their needs  Improve peer-to-peer relationships;  No travel time;  Fosters collaborative working;  Getting people from different practices in the same space;  More regular updates possible;  Linking different sites (with and without IRIS) in the same region possible;  Remote support empowers women to be safer. |

## Matrix c. Framework analysis of interviews – Clinicians

| Barriers  Uncertainty around the foreseeable future;  Not knowing remotely if it’s safe to ask;  GPs can forget parts of the training if they don’t apply it (so refreshers needed);  Patient/Service user’s restrictions at home (perpetrator more often present in house / Perpetrator using lockdown as a control/isolation tactic). | Enablers  Concise and informative training on DA;  Easy and quick process to get support for patients;  Ability to bring women to practice if unsure they are safe;  Non-confrontational approach to DA;  Systematic training (including refreshers);  Embedded AE;  Ensuring IRIS programme is running to best of its ability in current circumstances;  Structured way of asking about DA. |
| --- | --- |
| Losses  Women can no longer walk-in to the practice and see the worker;  Lack of human contact;  Missing women who it’s not safe to support now;  Not seeing body language, hearing tone of voice. | Benefits  Patients feel benefit from phone support;  IRIS programme provides named AE for direct referrals (benefit across the board);  GPs are better able to deal with cases of DA (remote and face-to-face) |

## Matrix d. Framework analysis of interviews – Service Users

| Barriers  No transition when support ends;  Difficulty believing to be in an abusive relationship;  Technical difficulties with online support program (Freedom Programme);  No link between the IRIS programme and the police. | Enablers  Clinician recognising DA;  Quick access to AE;  Using text and WhatsApp;  Health visitor linked with IRIS worker;  Access to multiple forms of support;  Easy access to online support  AE very responsive;  More frequent contact since lockdown |
| --- | --- |
| Losses  Clinicians and AEs lose ability to read body language and facial expression;  Physical contact with AE;  Some services not available in online form. | Benefits  Less personal (remote) – easier;  Easier not to miss appointments if in low mood (as they are remote) |
